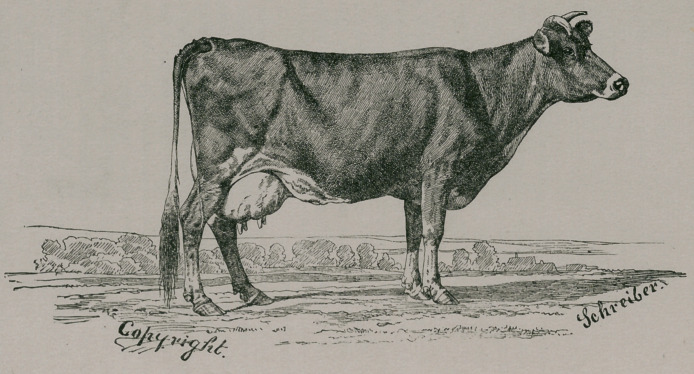# The Physiological Aspects of Breeding

**Published:** 1881-07

**Authors:** Allen S. Heath


### THE PHYSIOLOGICAL ASPECTS OF BREEDING.

---

EDITOR OF THE JOURNAL OF COMPARATIVE MEDICINE:—

Both anatomical and physiological characteristics must determine the virtues of animals bred for special purposes. Growth and repair of the animal body are made at the expense of food consumed; and the capability of the animal to digest and assimilate largely in excess of the simple requirements for growth and repair, renders such animal profitable in the peculiar products for which it is bred.

It is therefore of no absolute importance that an animal bred for any special purpose is a small consumer of food, for an animal that will profitably convert the largest amount of raw material into any desired product, with the least possible wear and tear, and also with the least useless expenditure of material, is the best, no matter how great the consumption of raw material.

Though all excellence for all purposes cannot be secured in the same individual, yet, it is desirable to add lesser qualities to that which is the chiefest aim for a special purpose. Desirable form, sound health, good constitution, with capacious and active digestive apparatus, are all essential in breeding for profit. All of these characteristics are dependent upon physiological laws; and he who would breed successfully for special products, must learn that upon formed character and proper development will hinge all hope of success.

Professor Morly Miles aptly says: "The principle of correlation that enables the breeder to determine the internal characteristics and tendencies of the organization, through the indications presented by the external form, is of general application, and may be made use of in the study of animals representing the different breeds; but the point upon which an opinion is formed will necessarily have a different value in each breed, from the difference in the qualities that constitute perfection."

The form and proportions of an animal indicates internal structure, and to some extent the functional activity of the internal organs, so that form and function are specially correlated in animals.

To secure this special development of form, with a corresponding activity of function, constitutes the science of legitimate breeding. The study of the external anatomy peculiar to the different breeds, and the study of the physiological phenomena of digestion, assimilation, glandular metabolism, etc., common to all, are indispensable to him who would breed with success.

As we purpose to illustrate the subject of breeding in general for different objects, we shall be as brief as possible in treating of the special animals which most profitably represent the different results of special breeding. The following illustration will convey the characteristics of external form and figure of the Jersey cow, "Favorite of the Elms" (1656), imported and owned by John T. Holly, of Plainfield, N. J.

To an expert, the form of this cow would indicate great excellence; but nothing but the positive test would show that in seven days this cow produced 16¼ pounds of butter of the finest quality.

This breed of cattle illustrates, very remarkably, to what perfection the art of breeding has been brought, as regards the production of a buttermaking animal. We see concentrated in it excellence for butter production far surpassing others of vastly greater bulk and weight. This is the result of long and persistent breeding for a special purpose; the steady application of physiological principles, the judicious union of the sexes of superior animals with a view to certain results.

In the Island of Jersey, which gave the name to this breed, so eminently suited to the butter-producing branch of the dairy, there are breeders of intelligence and judgment, who challenge even professional admiration in the means and in the results produced in perfecting the Jersey breed.

The development of the Jersey cow into so perfect a butter machine shows very clearly the means by which the stock of the country may be readily and certainly doubled in value. This is a subject of great interest to scientific and practical agriculture, and it foots up in a commercial point of view, thus attained, by millions of dollars. Such results can be accomplished only by studying the laws which govern animal life.

ALLEN S. HEATH, M.D.

## Figures and Tables

**Figure f1:**